# The evolving art of surgery for anterior fossa meningiomas

**DOI:** 10.3171/2025.10.FOCVID2562

**Published:** 2026-01-01

**Authors:** Takeo Goto, Carolina Benjamin, Wenya Linda Bi, Sébastien Froelich, Juan C. Fernandez-Miranda, Ian F. Dunn

**Affiliations:** 1 Department of Neurosurgery, Osaka Metropolitan University Graduate School of Medicine, Osaka, Japan;; 2 Department of Neurological Surgery, University of Miami, Florida;; 3 Department of Neurosurgery, Mass General Brigham, Boston, Massachusetts;; 4 Department of Neurosurgery, Hôpital Lariboisière, Paris, France;; 5 Department of Neurosurgery, Stanford University, Palo Alto, California; and; 6 Department of Neurosurgery, University of Oklahoma College of Medicine, Oklahoma City, Oklahoma

Anterior fossa meningiomas may be challenging tumors whose management continues to inspire considerable constructive debate. More than a century of experience with tumors in this region has established surgery as a clinically durable method of managing these tumors in patients in whom total removal is desired.

In approaching tumors, neurosurgeons must believe fully in the principles of microsurgery and bimanual microdissection, as well as in the critical role the arachnoid plays in enabling removal. The highly congested neurovascular anatomy of the region may be distorted by tumor of highly variable consistencies emanating from the anterior fossa dura, with degrees of hyperostosis and, in some cases, paranasal sinus entry. As these tumors grow, they may displace or encase the optic nerves or chiasm, and insinuation into one or both optic canals must be presumed so that the surgical approach allows for appropriate canal exploration. All canal quadrants, not just the medial corridor, may be involved. The relationship to the carotid arteries, their bifurcation, and the anterior cerebral branches, as well as corresponding lenticulostriate arteries, must be well understood; in some cases, the posterior circulation is also involved. Attempts to preserve the olfactory tracts in patients without preexisting anosmia should be pursued. The operative approach needs to enable access to secondarily involved fossae; the ability to handle intraoperative complications, especially vascular ones, if they arise; access to the full extent of the infiltrated dura to achieve a meaningfully complete resection; and reconstruction of tumor-inflicted or iatrogenic defects.

Adjunctive maneuvers in such approaches may include optic canal unroofing, anterior clinoidectomy, carotid dural ring dissection, pretemporal extradural dissection, posterior clinoidectomy, and anterior petrosectomy, among other a la carte selections. Unlocking the cavernous sinus and carotid control and even bypass, as one of the cases in this issue of *Neurosurgical Focus: Video* demonstrates, may be part of the operative deliberation.

The continued evolution and promulgation of endoscopic endonasal surgery, as well as other transcranial approaches via smaller incisions, has expanded the approach arsenal considerably, and there is emerging experience with fluorescence-guided surgery and exoscopic visualization. There are generally four categories of surgical approach used today—conventional craniotomy (e.g., pterional, bifrontal), open skull base (e.g., supraorbital, cranio-orbitozygomatic), minimally invasive transcranial (e.g., eyebrow, keyhole, transorbital), and endonasal endoscopic skull base approaches—and, as illustrated in this compendium, variable permutations and even serial or parallel combinations. Tumor, dura, and bone all may be removed through these approaches, with varying degrees of vascular control and access to the optic apparatus. Careful bimanual microdissection is critical. Ever-expanding endonasal approaches to anterior fossa meningiomas have proliferated against the backdrop of the successful application of transcranial approaches to these lesions. The core principles of endonasal approaches, which are fundamentally skull base approaches, include maximal bone removal for exposure, avoidance of brain retraction, preparation for reconstruction, bimanual dissection, and control of the carotid artery in its paraclival or cavernous segments. The tumor may be positioned between the surgeon and the optic apparatus, a highly desirable anatomical perspective that can facilitate gentle dissection and visual preservation. As the videos herein demonstrate, the medial and inferior optic canal can be beautifully addressed endonasally. The comparative advantages and disadvantages of open and endonasal approaches have been well discussed.

As the approach arsenal evolves, it is important that the neurosurgeon have a historical and innovative perspective, mastering the traditional approaches to anterior fossa meningiomas while appreciating the full spectrum of endonasal and minimally invasive approaches, to allow tailoring of the approach to the individual tumor and patient. Although multiple factors enter the decision of which operative approach to choose (e.g., tumor characteristics and direction of extent, the nature of the optic apparatus involvement, vascular relationships), should there be equipoise regarding the optimal strategy, a surgeon’s fundamental comfort level with the respective approaches should be the ultimate determining factor. What has emerged is the "right approach for the right tumor," without altering the goals of treatment. Whether the view is provided by a microscope, endoscope, or exoscope, fine and careful surgery through the application of bimanual microdissection continues to be fundamental to achieving complete tumor resection, deploying variations of the approaches described herein in the context of our enhanced biological understanding of the tumors themselves.

## Disclosures

Dr. Fernandez-Miranda reported personal fees from KLS Martin and nonfinancial support from Medtronic and Stryker outside the submitted work.

